# High Performance of a Metal Layer-Assisted Guided-Mode Resonance Biosensor Modulated by Double-Grating

**DOI:** 10.3390/bios11070221

**Published:** 2021-07-03

**Authors:** Chengrui Zhang, Yi Zhou, Lan Mi, Jiong Ma, Xiang Wu, Yiyan Fei

**Affiliations:** Department of Optical Science and Engineering, Shanghai Engineering Research Center of Ultra-Precision Optical Manufacturing, Key Laboratory of Micro and Nano Photonic Structures (Ministry of Education), School of Information Science and Technology, Fudan University, Shanghai 200433, China; 19210720089@fudan.edu.cn (C.Z.); 18110720008@fudan.edu.cn (Y.Z.); lanmi@fudan.edu.cn (L.M.); jiongma@fudan.edu.cn (J.M.); wuxiang@fudan.edu.cn (X.W.)

**Keywords:** sensors, guided-mode resonance, figure of merit, grating

## Abstract

Guided-mode resonance (GMR) sensors are widely used as biosensors with the advantages of simple structure, easy detection schemes, high efficiency, and narrow linewidth. However, their applications are limited by their relatively low sensitivity (<200 nm/RIU) and in turn low figure of merit (FOM, <100 1/RIU). Many efforts have been made to enhance the sensitivity or FOM, separately. To enhance the sensitivity and FOM simultaneously for more sensitive sensing, we proposed a metal layer-assisted double-grating (MADG) structure with the evanescent field extending to the sensing region enabled by the metal reflector layer underneath the double-grating. The influence of structural parameters was systematically investigated. Bulk sensitivity of 550.0 nm/RIU and FOM of 1571.4 1/RIU were obtained after numerical optimization. Compared with a single-grating structure, the surface sensitivity of the double-grating structure for protein adsorption increases by a factor of 2.4 times. The as-proposed MADG has a great potential to be a biosensor with high sensitivity and high accuracy.

## 1. Introduction

The advanced analytical biosensors are widely used as molecules detection and diagnostic tools, which are able to probe the interactions between chemical and biological molecules with high sensitivity and have found extensive applications in disease diagnosis, drug development, environmental pollution monitoring, and food safety detection [1,2,3]. The emergence of nanotechnology and nanofabrication has given rise to a variety of label-free biosensor technologies, such as cladding-mode resonance biosensors based on short- or long-period fiber gratings [4], Mach–Zehnder interferometer biosensors [5,6], surface plasmon resonance (SPR) biosensors [7,8,9], and guided mode resonance (GMR) biosensors [10,11,12,13,14,15].

GMR refers to the resonance between the incident light modulated by the grating and the conduction mode of the waveguide, and the GMR effect is widely used in the sensing field due to the advantages of simple structure, easy detection schemes, high efficiency, and narrow linewidth [12]. However, GMR sensors typically have relatively low sensitivity (<200 nm/RIU) and a small figure of merit (FOM, <100 1/RIU), which is defined as the sensitivity of the sensor divided by the full width at half maximum (FWHM) of the resonance (Sensitivity/FWHM) [16,17,18,19,20]. Biosensors with large sensitivity and FOM are more desirable since a large signal noise ratio is achievable for accurate detection of small signals during biosensing [21,22,23]. Many research groups have proposed several ways to improve the sensitivity of GMR sensors. Lu et al. proposed a compound waveguide grating biosensor via a modulated wave vector to enhance the sensitivity up to 345 nm/RIU, which is two times higher in magnitude than the normal case [18]. Wan et al. designed an ultralow refractive index porous silicon dioxide structure to make the resonance mode reside mainly in the sensing medium, which resulted in a sensitivity of up to 546 nm/RIU. In addition, metal structures are also used to improve the sensitivity of the sensor [24]. Lin et al. utilized a metal layer-assisted guided mode resonance (MaGMR) sensor to make the evanescent wave distribute asymmetrically in the waveguide layer which resulted in distribution of more electric field intensity in the analytes. The sensitivity of such a structure is 338.5 nm/RIU, increased by a factor of 1.5 over conventional structures [17]. Wang et al. proposed a hybrid guided-mode resonance/surface plasmon resonance structure to increase sensitivity to 1087 nm/RIU with a resonance wavelength of 1796.1 nm [25].

These reports demonstrated obvious sensitivity enhancement. However, the resonant linewidth also broadened (>10 nm) and the resultant FOM values were still small (~20 1/RIU). Some research groups also devoted their attention to improving the FOM values through narrowing the resonance linewidth without special efforts to increase sensitivity [13,15,26]. Lan et al. proposed a all-dielectric nano-silt arrayguided mode resonance sensor, and ultra-high FOM (~12,000 1/RIU) values were achieved with sensitivity in the range of 240 nm/RIU [26].

In this work, we proposed a GMR sensor with simultaneous enhancement of sensitivity and FOM. We explored a metal layer-assisted double-grating (MADG) structure-based GMR which consists of a substrate, a metal reflector and a double dielectric grating with two ridges in a period. With numerical simulation, we demonstrated that the bulk sensitivity of 550.0 nm/RIU and FOM of 1571.4 1/RIU can be achieved simultaneously. The proposed MADG-based GMR sensor has great potential in biosensing applications which require high sensitivity and a low detection limit.

## 2. Optimization of MADG Structure

### 2.1. MADG Structure

**Figure 1:**
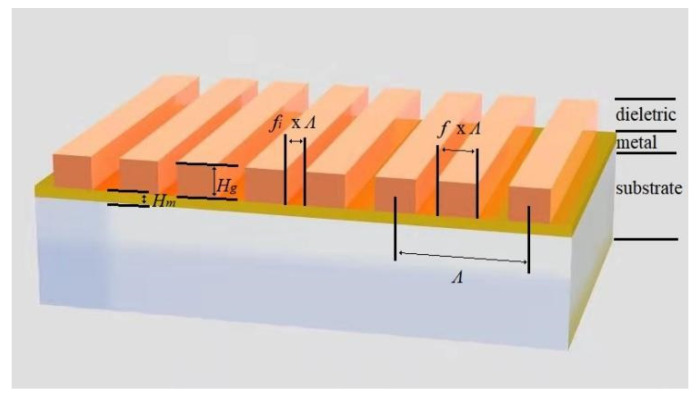
Configuration of MADG structure.

The proposed MADG structure that excites GMR is shown in Figure 1. On top of the SiO_2_ substrate, there is a metal reflector layer (Au) which is covered with a dielectric layer (HfO_2_, *n_g_* = 1.95) [27]. The metal reflector layer thickness is *H_m_*. The dielectric layer is designed to be a double grating which is composed of two ridges with identical width in a period. With a grating period of *Λ* and fill factors *f* and *f_i_*, the width of each ridge is *f* × *Λ*, the interval between two ridges is *f_i_* × *Λ*, and the interval between two periods is (1 − 2*f* − *f_i_*) × *Λ*. The depth of the grating is *H_g_*. The refractive index of the top medium surrounded the MADG structure is *n_a_*. The resonant features and electric field distribution of the MADG-based GMR structure were simulated with COMSOL Multiphysics 5.2a (COMSOL Inc., Stockholm, Sweden) [28].

### 2.2. Influence of f_i_ on MADG Based GMR Sensor Performance

**Figure 2:**
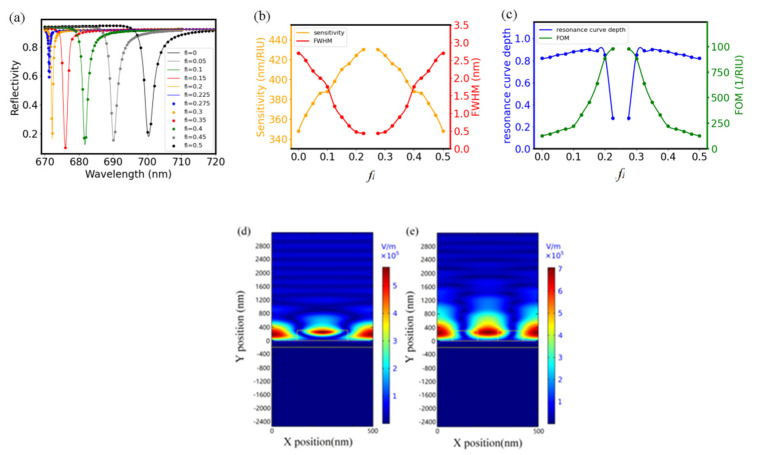
(**a**) Reflection spectrum of MADG based GMR sensor with *f_i_* ranging from 0 to 0.5 for the normal incident TE polarized light (electric field perpendicular to the plane of incidence light with normal direction of interface). (**b**) Dependence of sensitivity and FWHM of MADG based GMR on *f_i_*. (**c**) Dependence of FOM and resonance curve depth on *fi*. (**d**) Electric field distribution of GMR with *f_i_* = 0 and with (**e**) *f_i_* = 0.2. For simulation, *Λ* was set at 500 nm, *H_g_* at 300 nm, *H_m_* at 100 nm, and fill factor *f* at 0.25.

With *Λ* being set at 500 nm, *H_g_* at 300 nm, *H_m_* at 100 nm and fill factor *f* at 0.25, we studied how the fill factor *f_i_* affected the MADG-based GMR’s performance. As shown in Figure 2a, the resonance wavelength is in the range of visible light, which demonstrates a red shift, with *f_i_* increasing from 0 to 0.225. When *f_i_* is close to 0.25, the resonance disappears. With *f_i_* increasing from 0.275 to 0.5, there are strong resonances again with features the same as resonances at 0.5 − *f_i_*.

Figure 2b shows that the sensitivity of the GMR sensor increases and FWHM decreases monotonically with *f_i_* less than 0.25. In addition, the sensitivity decreases and FWHM increases monotonically with *f_i_* larger than 0.25. The sensitivity and FWHM are not shown for *f_i_* = 0.25 where resonance disappears. As demonstrated in Figure 2c, the FOM of the MADG-based GMR sensor maximizes with *f_i_* close to 0.25, similar to the behavior of the sensitivity.

Figure 2d,e show the electric field distribution of the MADG-based GMR structure with *f_i_* = 0 and *f_i_* = 0.2, respectively. Due to the metal layer underneath the grating, no electric field distributes in the substrate and the evanescent diffraction field distributes mostly in the top medium for sensing. Around the grating ridge, most of the electric field is located inside the ridge with *f_i_* = 0, while it is mostly distributes in the top medium between two ridges with *f_i_* = 0.2. In addition, the penetration depth of the evanescent diffraction field is deeper with *f_i_* = 0.2. The distribution of the electric field in the top medium and the deep penetration depth with *f_i_* = 0.2 demonstrate that the MADG-based GMR sensor provides more evanescent energy for sensing, which may be responsible for the improvement in the sensitivity with the double grating structure [29]. The larger overlap area between analytes and evanescent diffraction field provided by the MADG structure has the potential to enhance the sensitivity of the techniques taking advantage of evanescent field, such as the plasmon-enhanced fluorescence method for the detection of molecules of various sizes [30,31].

The change in FWHM with *f_i_* can be attributed to the coupling changes between evanescent diffraction fields and the leaky guided modes. The permittivity of the periodic grating can be expanded into Fourier series [32],

(1) $$\varepsilon =\underset{n}{\sum }\varepsilon_{n}exp\left(i\frac{2n\pi x}{\Lambda }\right)$$

where the Fourier harmonic coefficients *ε_n_* can be expressed as,

(2) $$\varepsilon_{0}=2fn_{g}^{2}+\left(1-2f\right)n_{a}^{2}$$

(3) $$\varepsilon_{n}=(n_{g}^{2}-n_{a}^{2})\frac{sin\left[n\pi \left(1-2f-f_{i}\right)\right]-sin\left(n\pi f_{i}\right)}{n\pi },\;\left(n=\pm 1,\pm 2,\ldots \pm N\ldots \right)$$

According to the rigorous coupled-wave theory [33], the Fourier harmonic coefficients *ε_n_* regulate the interaction among evanescent diffraction fields and the leaky guided modes [30]. Since the MADG-based GMR sensor excites leaky guided modes through the first evanescent diffracted order of the grating, the coupling between the evanescent diffraction fields and the leaky guided modes is mainly determined by *ε*_1_. Equation (3) shows that |*ε*_1_| decreases to zero when *f_i_* changes from 0 to 0.25 and |*ε*_1_| increases from zero with *f_i_* changing from 0.25 to 0.5. Smaller |*ε*_1_| signifies poorer coupling, causing decreased spectrum linewidth [32], which may explain behaviors of FWHM. 

One more feature in Figure 2a is the depth of the resonance curve, which is defined as reflectivity at the inflection point minus the reflectivity at the resonance point. Figure 2c shows that the resonance curve depths are close to 0.8 with *f_i_* less than 0.2 and decrease sharply to around 0.1 with *f_i_* changing from 0.2 to 0.25. Since resonance signals with larger depth have better noise tolerating capability for better sensing performance, *f_i_* = 0.2 is used in the following simulations with which large sensitivity, large FOM and large resonance curve depth are all achievable.

### 2.3. Influence of H_m_ on MADG Based GMR Sensor Performance

**Figure 3:**
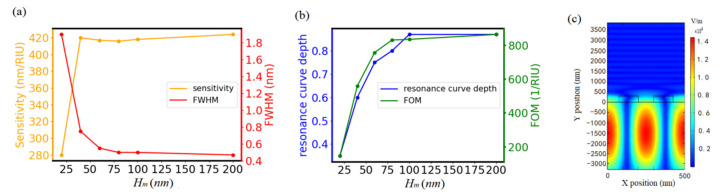
(**a**) Dependence of sensitivity and FWHM on *H_m_*. (**b**) Dependence of FOM and resonance curve depth on *H_m_*. (**c**) Electric field distribution of GMR with no metal reflector layer. For simulation, *Λ* was set at 500 nm, *H_g_* at 300 nm, fill factor *f* at 0.25, and *f_i_* at 0.2.

With *Λ* at 500 nm, *H_g_* at 300 nm, *f* at 0.25, and *f_i_* at 0.2, we studied the influences of the metal reflector layer underneath the double grating on performance of GMR sensor. As shown in Figure 3a,b, the sensitivity increases and the FWHM decreases with *H_m_*, which results in FOM increasing with *H_m_*. The FOM almost levels off when *H_m_* is larger than 100 nm, meaning that *H_m_* = 100 nm is used in the following simulations. In addition, the resonance curve depth is close to 0.9 with *H_m_* = 100 nm which provides good noise tolerating capability. 

To understand the increase in sensitivity with *H_m_*, Figure 3c shows the electric field distribution of GMR without the metal reflector layer. Compared with the electric field distribution with the metal reflector layer shown in Figure 2e, most of the electric field distributes in the substrate layer rather than in the analytes when there is no metal reflector layer. The increase in sensitivity from 15 nm/RIU with *H_m_* = 0 nm to 420 nm/RIU with *H_m_* = 100 nm could be due to the asymmetrical evanescent diffraction field wave distribution in the waveguide layer and the distribution of more electric field intensity in the analytes [19].

### 2.4. Influence of Λ and H_g_ on MADG Based GMR Sensor Performance

**Figure 4:**
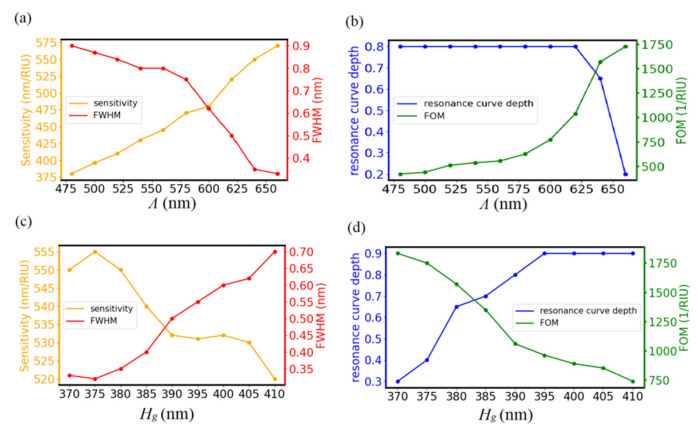
(**a**) Dependence of sensitivity and FWHM (**b**) resonance curve depth and FOM on *Λ* with *H_g_* at 380 nm, *H_m_* t at 100 nm, *f* at 0.25, and *f_i_* at 0.2. (**c**) Dependence of sensitivity and FWHM (**d**) resonance curve depth and FOM on *H_g_* with *Λ* at 640 nm, *H_m_* at 100 nm, *f* at 0.25, and *f_i_* at 0.2.

Figure 4a,b show the dependence of GMR performance on *Λ* with Hm at 100 nm, *f* at 0.25, and *f_i_* at 0.2. To obtain resonance curves under phase matching conditions with *Λ* ranging from 480 to 660 nm, Hg was set at 380 nm. It is clear that a large *Λ* provides high sensitivity and small FWHM, which is similar to the previous studies [34,35]. Even though both sensitivity and FOM monotonically increase with *Λ* within the range of simulations, resonance curve depths drop dramatically with *Λ* at 660 nm. In this case, *Λ* = 640 nm is used for following simulations. In addition, the resonant wavelength at *Λ* = 640 nm is 858.2 nm, which makes the as-designed sensor a promising candidate for applications with high sensitivity and relatively low absorption in water.

Figure 4c,d show the dependence of GMR performance on grating *H_g_* with *Λ* at 640 nm, *H_m_* at 100 nm, *f* at 0.25, and *f_i_* at 0.2. In order to satisfy the phase matching condition with *Λ* at 640 nm, *H_g_* varies from 370 to 410 nm. Both sensitivity and FOM decrease with *H_g_* and maximal FOM is located at 370 nm, at which the resonance curve depth is only 0.3. To obtain large sensitivity and FOM with acceptable resonance curve depth, *H_g_* = 380 nm was used for following simulations.

### 2.5. Bulk and Surface Sensitivity

**Figure 5:**
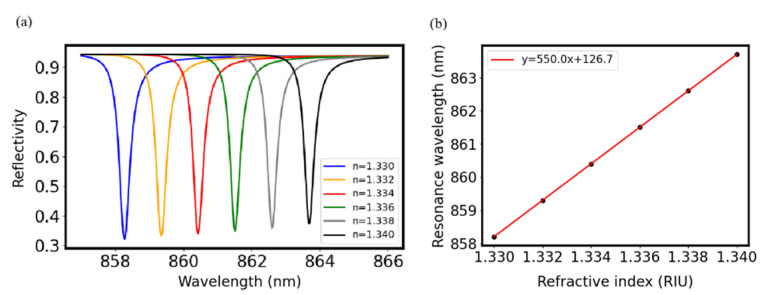
(**a**) Reflection spectra of the MADG-based GMR sensor immersed in liquids with increasing refractive indices. (**b**) Resonance wavelength of the MADG-based GMR sensor changes as a function of the liquid refractive index. For simulation, *Λ* was set at 640 nm, *H_g_* at 380 nm, *H_m_* at 100 nm, *f* at 0.25, and *f_i_* at 0.2.

With *Λ* at 640 nm, *H_g_* at 380 nm, *H_m_* at 100 nm, *f* at 0.25, and *f_i_* at 0.2, the MADG-based GMR sensor was immersed in liquids with increasing refractive indices. Figure 5a,b show that the resonance wavelength increases linearly with refractive index and the slope (i.e., bulk sensitivity) is 550.0 nm/RIU. With FWHM being 0.35 nm, FOM is calculated to be 1571.4 1/RIU. Comparing with other GMR sensors, the MADG-based GMR sensor provides both high sensitivity and a large FOM, as shown in Table 1.

**Figure 6:**
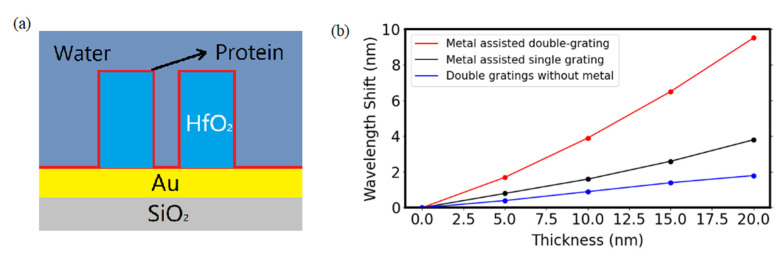
(**a**) Protein adsorption on the MADG-based GMR sensor. (**b**) The shift of resonant wavelength as a function of the protein thickness on GMR sensor. *Λ* was set at 640 nm, *H_g_* at 380 nm, *H_m_* at 100 nm, *f* at 0.25, and *f_i_* at 0.2.

We then investigated the surface sensitivity with the protein sample adsorbing on the MADG-based GMR sensor. For simulation, we assumed a refractive index of 1.5 [36] and the protein thickness increasing from 0 nm to 20 nm to calculate the surface sensitivity [37]. Figure 6b shows the resonant wavelength of GMR as a function of the protein thickness. The sensitivity of MADG with double grating is 0.415 nm/nm, which is 2.4 times of that of metal assist single grating and 5.5 times of that of double grating without metal layer.

## 3. Results

In summary, a metal layer-assisted guided mode resonance biosensor modulated by a double grating structure was designed to achieve both high sensitivity and high FOM for optical biosensing. The sensitivity is increased by the metal reflector under the double grating layer, which causes asymmetrical electric field distribution and leads to longer penetration depth and larger overlap area between the analytes and the evanescent diffraction field. The spectral linewidth is optimized through double grating by modulating coupling between the evanescent diffraction fields and the leaky guided modes. With optimization, the MADG-based GMR sensor is able to provide bulk sensitivity of 550.0 nm/RIU and FOM of 1571.4 1/RIU, which shows great potential for sensitive label-free biosensing.

## Figures and Tables

**Table 1:** Sensitivity, FOM, and resonance wavelength of some GMR sensors.

GMR Sensors	Sensitivity (nm/RIU)	FOM (1/RIU)	Wavelength (nm)	Reference
MADG based GMR device	550.0	1571	858.2	This work
One-dimensional all-dielectric nano-slit array	240	12,000	819	[26]
Hybird GMR/SPR sensor	1087	23	1796.1	[25]
Compound waveguide grating	345	17.3	1580	[18]
Ultralow RI substrate GMR	546	~273	~784	[24]

## Data Availability

Not applicable.
